# A Hybrid Tracking System of Full-Body Motion Inside Crowds

**DOI:** 10.3390/s21062108

**Published:** 2021-03-17

**Authors:** Maik Boltes, Juliane Adrian, Anna-Katharina Raytarowski

**Affiliations:** Institute for Advanced Simulation 7, Civil Safety Research, Forschungszentrum Jülich, 52428 Jülich, Germany; j.adrian@fz-juelich.de (J.A.); a.raytarowski@fz-juelich.de (A.-K.R.)

**Keywords:** pedestrian dynamics, crowd, camera tracking, motion capturing, IMU, sensor fusion

## Abstract

For our understanding of the dynamics inside crowds, reliable empirical data are needed, which could enable increases in safety and comfort for pedestrians and the design of models reflecting the real dynamics. A well-calibrated camera system can extract absolute head position with high accuracy. The inclusion of inertial sensors or even self-contained full-body motion capturing systems allows the relative tracking of invisible people or body parts or capturing the locomotion of the whole body even in dense crowds. The newly introduced hybrid system maps the trajectory of the top of the head coming from a full-body motion tracking system to the head trajectory of a camera system in global space. The fused data enable the analysis of possible correlations of all observables. In this paper we present an experiment of people passing though a bottleneck and show by example the influences of bottleneck width and motivation on the overall movement, velocity, stepping locomotion and rotation of the pelvis. The hybrid tracking system opens up new possibilities for analyzing pedestrian dynamics inside crowds, such as the space requirement while passing through a bottleneck. The system allows linking any body motion to characteristics describing the situation of a person inside a crowd, such as the density or movements of other participants nearby.

## 1. Introduction

A better understanding of crowd dynamics is crucial in order to increase safety and comfort for pedestrians, which might result in improved escape routes and optimized transport infrastructures. Quantifications in guidelines, construction manuals and legal regulations as well as the design, calibration and verification of microscopic models need a detailed analysis of crowd motion. For this analysis, empirical data, such as the trajectory of every single person with high spatial and temporal resolution inside a crowd, are needed [1,2].

Experiments under laboratory conditions investigating pedestrian streams enable the study of selected parameters under well-defined constant conditions independently of undesired influences. For controlled experiments, parameters of interest can be varied as needed and may also be adjusted to situations which rarely occur in field studies, e.g., very high densities [3] or a specific quantity of disabled people [4]. Such experiments allow the collection of exact positions and additional characteristics for each participant, such as age, gender, size, fitness and cultural origin. Furthermore, controlled experiments enable the measurements of quantities, giving conclusions on psychological aspects [5,6] that are impossible to survey by pure observation.

High accuracy of extracted data is ensured by optimal conditions in artificial environments [7]. The high data quality enables the development of measurement methods providing high resolution in time and space of quantities such as density, flow and velocity with small scatter [8,9]. Even the influences of parameters with minor impacts, such as body height, gender and age, on stepping locomotion or interpersonal space [10,11], can be studied using high quality trajectories [12] enriched with additional data.

Different levels of detail of the information extracted from laboratory experiments and from field studies can be found in the literature. The detection of single pedestrians is not necessary for the calculation of the velocity in a certain area or the estimation of the density [13,14]. The optical flow can be used to get an impression of the overall movement of a crowd, separate the crowd in different areas depending on the activity in that area or detect abnormal behavior [15,16,17]. Nevertheless, for a high level of detail, detection and tracking of the skeleton of a person is invaluable. Back in the 19th century, the first studies analyzing precise motion sequences started with the application of the chronophotography [18]. Nowadays, skeleton detection and tracking is performed automatically in real-time, e.g., with optical motion capturing (MoCap) systems for film production, steering virtual characters, or by using Microsoft Kinect for gaming [19].

For the analysis of peoples’ motion, the highest possible level of detail is favorable, which is achieved by tracking the full locomotive system of each subject. However, for a high-density crowd, optical systems fail because the lines of sight between camera and the body parts to be tracked are hidden by other persons or body parts. Therefore, most experimental data provided for analysis and modeling pedestrian dynamics are limited to trajectories of the head of every single pedestrian [20,21,22,23,24,25,26,27,28,29,30,31]. Only some datasets include additional global (e.g., distribution of gender or age) or individual (e.g., body size, and head or shoulder orientation) information [32]. The lack of more detailed data is one reason why models simulating pedestrian dynamics do not consider the motion of all body parts, even though taking, e.g., the gait of bipedal movement, the step length or the orientation of the body with respect to the main movement direction into account would enhance the quality of simulation results [33,34,35,36].

To enable the analysis of the influence of full-body motion on pedestrian dynamics and to overcome the problem of occlusion of optical tracking systems in crowds, systems based on inertial measurement units (IMU) can be used (business solutions: e.g., Xsens [37], Perception Neuron [38] and SST Systems [39]). The functionality and possibilities, but also the limitations of such systems, are briefly described in Section 2.1. Other techniques without the need of a free line of sight are magnetic systems, which are very sensitive to the presence of metal objects and electrical sources; and mechanical systems, which use a skeletal-like structure that users have to wear [40], which would significantly impair the movement in a crowd.

Besides applications for motion picture or video game production, most research applications using MoCap systems belong to the field of biomechanics and studies with a medical focus [41,42]. Furthermore, there are several applications of inertial MoCap systems related to safety in (autonomous) vehicular traffic studies (e.g., [43,44]). Ref. [43] combined the Xsens MoCap system with virtual reality and used a dynamic driving simulator. They investigated human crossing behavior without the risks of real pre-crash scenarios. Ref. [44] employed the Xsens MoCap system as a reference for limb movements in, e.g., road crossing scenarios. They compared the limb movements obtained by the MoCap system with Doppler radar data, which can potentially be obtained by a car in its near environment. Other applications are related to indoor navigation. Ref. [45], e.g., compared the joint angle and step length data that they obtained by using different IMU sensors attached to the lower body.

IMU-based systems measure relative movement and are not able to track the absolute position accurately for a longer time [46]. Thus, for studying the dynamics inside crowds and the interactions between people and their environments, an additional absolute positioning system is needed. Inside buildings, global positioning systems (GPS) cannot be used. Map information as an additional constraint can help to limit the drift, but does not give highly accurate positional data [47]. Additionally, other techniques using radio frequency identification, ultrasonic sensors, wireless local area networks or Bluetooth beacons are less precise than ultra wideband or image-based technologies [48]. For controlled experiments, people and the environment can be equipped with supporting techniques or markers, and critical occlusion can be prevented by extending the system. Ultra wideband (UWB)-based time of arrival (TOA) ranging is an often used, high-precision localization technology with a measurement accuracy reaching the centimeter level [49,50], but it is less precise than an image-based system using markers can be. According to Teixeira [51], the best modality across the board for detection and tracking of people is vision (e.g., cameras or other imagers).

For experiments with up to a thousand participants [52], a camera-based system can extract highly accurate positional data from each participant [7]. Thus, the absolute positions for our studies on pedestrian dynamics and for the proposed method are provided by a well-calibrated overhead camera grid [7]. For the detection and tracking of marked heads within image sequences, a lot of well established methods with low error exist (e.g., mean-shift and particle filter [53,54]). For the structured marker we use for detection the shape of oriented isolines of the same brightness, and for tracking the iterative Lucas Kanade feature tracker [55]. For field studies the task of detection and tracking is much harder and has to deal, e.g., with unequipped people and occlusion [56,57].

By using two systems as a synchronized combination of an inertial and an optical system, we set up a hybrid tracking system (see Section 2.2). The resulting fused dataset enables 3D motion capturing of full-body motion in crowds, enabling studying, e.g., the relation between the location in front of a bottleneck to the rotation of the pelvis at that position (see Section 3.1).

## 2. Methods

### 2.1. IMU-Based 3D Motion Capturing

An IMU is a combination of sensors which allows one to measure the relative motion of an object. Such an IMU consists of an accelerometer, a gyroscope and a magnetometer measuring the acceleration, angular rate and magnetic field. Some IMUs also measure the air pressure for height calculation. With those readings it is possible to keep track of relative changes of a pedestrian’s position. Starting with an initial position, the next position is calculated by applying algorithms from the fields of inertial navigation or step-and-heading systems such as double integration of the acceleration.

By attaching multiple sensors to a person’s body, tracking of the 3D full-body motion as in [58,59,60] is possible. By using multiple IMUs attached to independently movable segments of a body, the full-body motion can be computed based on constraints coming from a biomechanical model of the human skeleton and with help of sensor fusion algorithms. The IMUs of such a system are self-contained and light weight, and if desired the data can be stored locally on the sensor and controlled wirelessly. No continuous connection or line of sight to an external device is needed. The data acquisition of the entire system is able to work self-sufficiently and the small IMUs do not restrict persons in their freedom of movement.

For the experiments presented in this paper, the system consisting of the hardware MVN Awinda, and software MVN Analyze from Xsens [37] was used in single level mode. The system comprised 17 IMUs, one for each distinctive segment of the human body. A detailed description of this system can be found in [59,61]. Every time a person was putting on the system or changed the position of a single sensor, a calibration process was performed as described in [62] to estimate the dimensions of the person being tracked, along with the orientations of the sensors with respect to the corresponding segments. After a stand-still phase in neutral position, the wearer of the system was asked to walk forwards and backwards in a normal fashion, thereby leaving and returning to his starting position. Quantities about the accuracy of the tracker and MVN fusion engine can be found in [62] (e.g., the dynamic accuracy of the tracker has a root mean square error of 1°).

The disadvantage coming from the self-sufficient construction using relative measurements is a positional drift which can compound and accumulate over time [46]. The longer the tracking time, the larger the drift so that the calculation of an absolute position leads to increasing errors. Kalman and particle filters can slow down the accumulation process [63], but cannot eliminate it completely.

**Figure 1:**
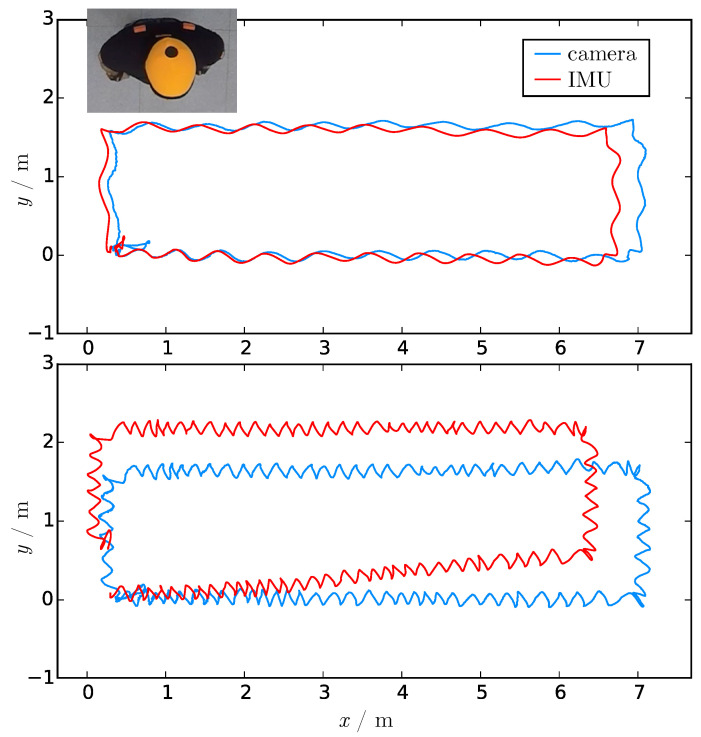
Trajectories of a person’s head walking along a rectangle with the same position being the start and the end. The blue path results from a camera system detecting and tracking a black dot on an orange cap. The red path is the head trajectory of the used IMU-based system which is superposed by a drift that increases with time. (**Top**) slow walking with 0.336 ms^−1^. (**Bottom**) very slow walking with 0.054 ms^−1^.

Figure 1 illustrates the drift of the IMU-based system. A person wearing an orange cap with a black dot is walking along a rectangle ending at the starting position.

The blue path is the trajectory of the black dot over time resulting from a camera-based tracking system [7]. It is the ground truth because of the very accurate calibration with an error of 1.5 cm when taking the height of the person into account, although the camera trajectory has some local errors due to the perspective distortion and the neglect of bobbing. Jittering would have been larger if instead of a structured marker, a cap without a black dot was used. An unsteady trajectory also occurs if the black dot is temporarily not visible, and resulted for our example in a jerk that can be seen in the bottom right of Figure 3.

The red path shows the trajectory of the top of the person’s head calculated from the IMU-based system. For the top figure, the person walked slowly with an average speed of 0.34 ms^−1^ for a duration of 50 s. For the bottom figure, the average speed was even slower at 0.05 ms^−1^, and it took 310 s to walk along the rectangular path. The drift was more prominent the longer the tracking was. The maximum error for the top was 0.35 m and for the bottom 0.87 m. The systematic error is somewhat compensated when the person moves back to the starting point.

### 2.2. Hybrid Tracking System

The camera tracking system has a small global error, but can only track visible body parts in two dimensions without bobbing. IMU-based MoCap systems have the opportunity to extract highly accurate relative 3D full-body motion even for non visible body parts, for instance, leg movement or pelvis rotation for persons inside a crowd.

**Figure 2:**
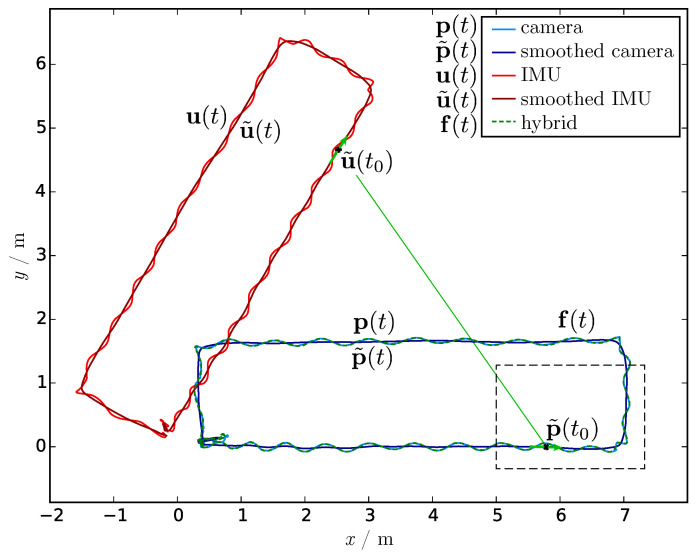
Trajectories of the top of Figure 1 from a person’s head walking along a rectangle, where the red IMU trajectory u is rotated by 60° for demonstration purposes only. The smoothed trajectories $\tilde{p}$ and $\tilde{u}$ are displayed darkened. The dashed green path f shows the resulting fused trajectory of the hybrid tracking system (HTS). The dashed region is enlarged in Figure 3 to show the rotation angle resulting from the main movement directions of the smoothed camera and IMU trajectory.

The main idea of the introduced hybrid tracking system (HTS) is the mapping of the trajectory of the top of the head u coming from a relative full-body motion tracking system to the head trajectory p of a camera system in global space (see Figure 2), thereby combining the best of both worlds. The global space is oriented so that the horizontal movement takes place in the *xy*-plane and the *z*-component reflects the height above the ground.

For showing the robustness of the presented fusion algorithm, a synthetic dataset was created by rotating the IMU data shown in the top of Figure 1 by 60°. Then, the proposed method was applied to the synthetic data.

To reduce the influence of local movement or jitter and to gain the main movement direction retaining the drift over time, both head trajectories are smoothed with the same method. A central moving average is used with an interval of 2 s so that the body swaying of the bipedal gait is eliminated also for slow movement. For advanced smoothing techniques, see [64]. As the *z*-component of the camera trajectory is only mapped to the height of the person, whereas the IMU trajectory includes the bobbing, the *z*-components of both smoothed trajectories are neglected and set to zero. The resulting trajectories are visualized in dark red ($\tilde{u}$) and dark blue ($\tilde{p}$) in Figure 2. The fusion method is not sensitive to the choice of the smoothing interval as long as the swaying is eliminated and both trajectories are smoothed with the same smoothing method and related parameters.

After smoothing the trajectories, the main movement directions of both trajectories were calculated. $r_{p}$ is pointing to the main movement direction of the camera trajectory and $r_{u}$ is pointing along the direction of the IMU trajectory. For time *t* the formulas are

(1) $$\begin{array}{cc} r_{p}\left(t\right) & =\tilde{p}\left(t+\Delta t\right)-\tilde{p}\left(t-\Delta t\right),\\ r_{u}\left(t\right) & =\tilde{u}\left(t+\Delta t\right)-\tilde{u}\left(t-\Delta t\right). \end{array}$$

Δt can be small (here 1 s), because jittering and swaying have already been eliminated, but for the stand-still phase, the minimum lengths of $r_{p}$ and $r_{u}$ should be larger than head movement during this period (here 1 m) so that a small error in the detected position does not noticeably affect the direction determination. As long as the datasets are well synchronized in time, the fusion algorithm is also robust regarding the parameter Δt. When a turning happens within the camera trajectory, turning occurs in the IMU trajectory.

The next step is the calculation of the rotation angle α between the main movement directions of the camera and IMU trajectory:

(2) $$\alpha \left(t\right)=\left\{\begin{array}{cc} \arccos(\frac{\langle r_{p}\left(t\right),r_{u}\left(t\right)\rangle }{∥r_{p}\left(t\right)∥∥r_{u}\left(t\right)∥}),\; & \mathrm{for}\left|\begin{array}{cc} r_{p_{x}}\left(t\right) & r_{ux}\left(t\right)\\ r_{p_{y}}\left(t\right) & r_{uy}\left(t\right) \end{array}\right|\geq 0\\ 2\pi -\arccos(\frac{\langle r_{p}\left(t\right),r_{u}\left(t\right)\rangle }{∥r_{p}\left(t\right)∥∥r_{u}\left(t\right)∥}),\; & \mathrm{for}\left|\begin{array}{cc} r_{p_{x}}\left(t\right) & r_{ux}\left(t\right)\\ r_{p_{y}}\left(t\right) & r_{uy}\left(t\right) \end{array}\right|<0 \end{array}\right..$$

**Figure 3:**
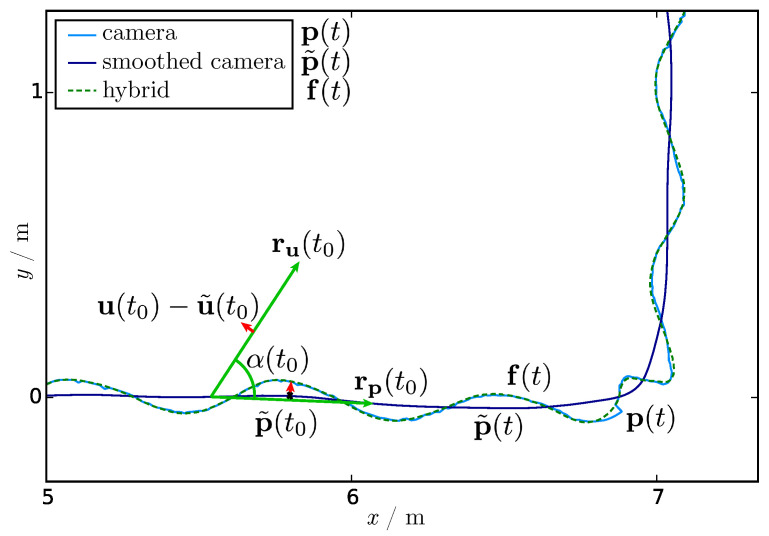
Enlarged cropped region of Figure 2 showing the rotation angle α between the main movement directions of the smoothed camera $r_{p}$, and the IMU trajectory $r_{u}$ visualized as a green arrow in each case. The red arrow shows the difference between the head trajectory of the IMU system u and the smoothed IMU trajectory $\tilde{u}$, whose rotated vector was added to the smoothed camera trajectory $\tilde{p}$ (dark blue path) to get the resulting fused trajectory f (green dashed path). The jerk in the camera trajectory in the bottom right came from a temporary loss of sight of the tracked black dot.

The case distinction according the determinant of the *x* and *y*-components of the main movement direction is needed for handling all angles up to a full angle. The angle is visualized in Figure 3, the enlarged dashed area of Figure 2.

The fused trajectory f of the hybrid tracking system finally is

(3) $$f\left(t\right)=\tilde{p}\left(t\right)+R\left(-\alpha \left(t\right)\right)\left(u\left(t\right)-\tilde{u}\left(t\right)\right),$$

where R(−α(t)) is the rotation matrix of the angle −α(t) within the xy-plane for rotating the difference between the head trajectory of the IMU system and smoothed IMU trajectory (red arrow in Figure 3). The result is a jitter-free head trajectory of the full-body motion, including bobbing within the camera coordinate system (see green dashed lines in Figure 2 and Figure 3). Jittering and other jerks have been culled, as shown, e.g., in the bottom right of Figure 3.

Different frame or sampling rates (fs${}^{-1}$) have to be taken into account. Here the camera system extracts trajectories with a frame rate of 25 fs^−1^, the IMU system calculates trajectories with a sampling rate of 60 fs^−1^ and the resulting trajectories have a frame rate of 60 fs^−1^. The mapping of intermediate values that do not match each other is done by linear interpolation. Thus for time *t* between successive frames, at time $t_{1}$ and $t_{2}$, the interpolated value is

(4) $$p\left(t\right)=p\left(t_{1}\right)+\frac{t-t_{1}}{t_{2}-t_{1}}\left(p\left(t_{2}\right)-p\left(t_{1}\right)\right).$$

In summary, for the HTS, this results in the following steps:

1. Smooth camera and IMU trajectories;
2. Calculate angle between main movement directions;
3. Add rotated difference of the head trajectory of the IMU system and smoothed IMU trajectory to the smoothed camera trajectory.

To move other points $u_{s}$ of the biomechanical model within the IMU coordinate system toward the camera coordinate system, the formula is as follows:

(5) $$f_{s}\left(t\right)=f\left(t\right)+R\left(-\alpha \left(t\right)\right)\left(u_{s}\left(t\right)-u\left(t\right)\right).$$

### 2.3. Errors

To fuse camera and IMU data, the mapping of corresponding persons in the datasets has to be ensured. The time synchronization of both systems has to be very accurate, as already mentioned. Inaccurate time synchronization leads to an incorrect fit. The distance between the original camera trajectory and the trajectory from HTS is a good indication of the error in time between the datasets. For a time offset of Δt, shifting u(t) to u(t+Δt) resulting in a fused trajectory $f_{\Delta t}\left(t\right)$ the distance is

(6) $$d_{\Delta t}\left(t\right)=∥p\left(t\right)-f_{\Delta t}\left(t\right)∥.$$

**Figure 4:**
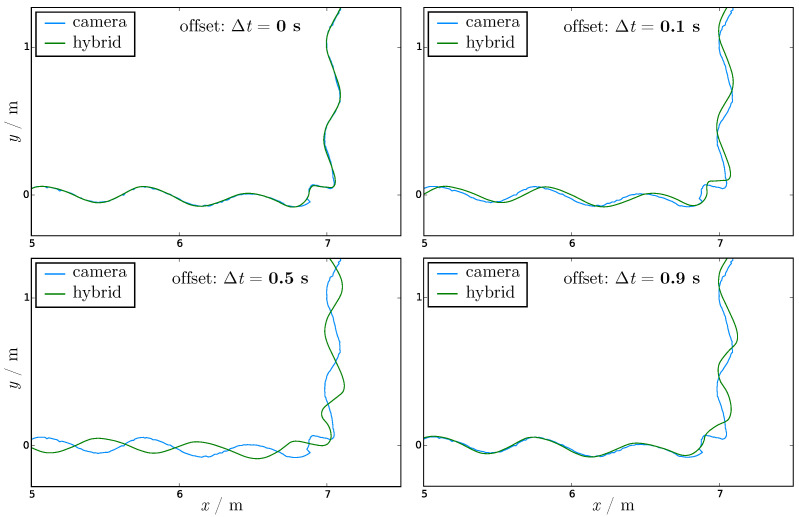
The camera trajectory in blue and the fused trajectory of the HTS in green for the same region as in Figure 3 for given time offset Δt values of 0 s, 0.1 s, 0.5 s and 0.9 s with regard to the correct synchronous time. The average distances $\bar{d_{\Delta t}}$ between the trajectories are listed in Table 1.

This distance should be as small as possible in the xy-plane. Figure 4 and Table 1 show and lists the time shift Δt and average distance $\bar{d_{\Delta t}}$ for the example also shown in Figure 3.

**Figure 5:**
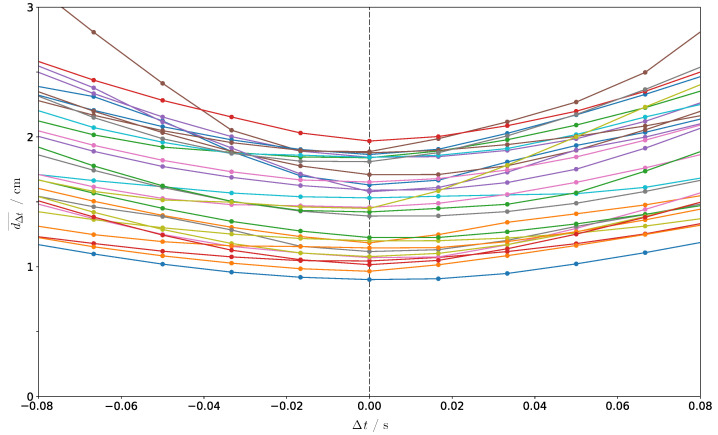
Average distance $\bar{d_{\Delta t}}$ between the original camera trajectory and the corresponding trajectory from HTS when shifting the IMU data by Δt around the minimum at correct time t=0 (known for these experiments). Each line represents an other experimental run and thus an independent fusion result.

The error according the time synchronization could be conversely used to fine-tune the time synchronization itself by looking for the time shift with the minimum in distance. Figure 5 shows for a series of experiments the average distance $\bar{d_{\Delta t}}$ when shifting the IMU data by Δt. The distance curves are convex functions with a stable minimum at correct time t=0 (known for these experiments).

Interframe interpolation may have to be used, especially to consider different frame rates. Already, a time offset of two hundredths of a second enlarges the average distance between the fused trajectory and the camera trajectory in the xy-plane for the example in Figure 4 by 19%. Even if the error decreases when shifting in time by the duration of a full stride length (here 0.9 s), the error is higher than for the correct time, because the stride duration may change over time and the person is not only going straight ahead.

**Figure 6:**
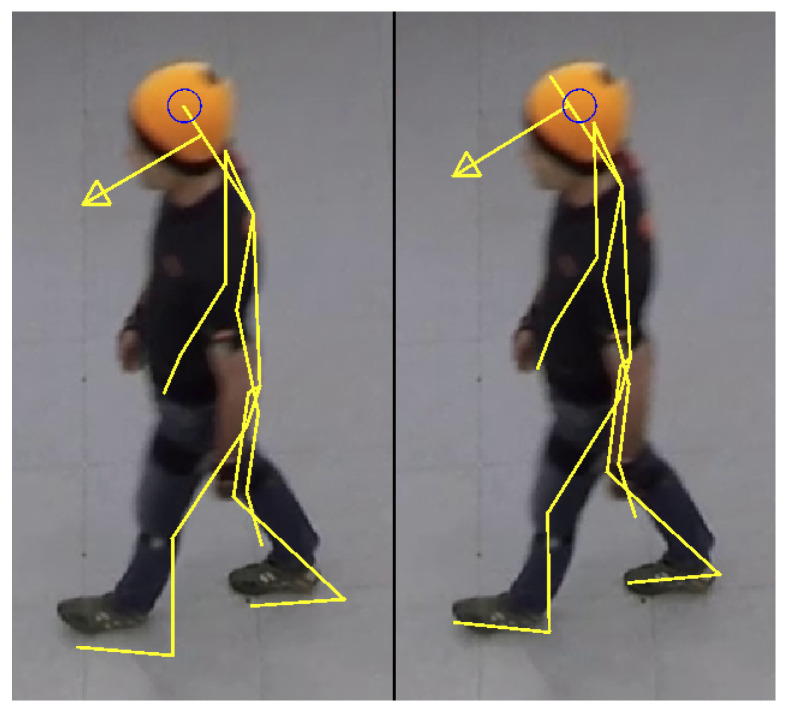
Snapshot of the person within the experiment from a slanted view and a projected line-based yellow overlay of the skeleton coming from the IMU system (arrow points in the direction of the head orientation). (**Left**) Center of gravity of the colored blob of the worn orange cap (center of blue circle) is chosen for the absolute position of the person’s head coming from the camera system. (**Right**) Position of the top of the head coming from the camera system is chosen according the underlying biomechanical model of the IMU system, which leads to a better fusion result.

The trajectory of the head coming from the camera system is assumed to be correct in global space. For this assumption, lens distortion, perspective distortion and the height of a person has to be taken into account [7,55]. The fused IMU trajectory is the trajectory of the top of the person’s head according the underlying biomechanical model. This leads to an important condition for the camera trajectory, which also has to represent the same top of head as described in section “Anatomical model” of [62]. Only using the center of gravity of a color blob of a worn cap (center of blue circle for the orange cap in Figure 6) leads to a changing and thus incorrect shifted position to the direction underneath the camera (left of Figure 6), caused by varying viewing angles [12]. However, also for a structured marker and thus getting a fixed point on a person’s head, one has to carefully select the correct position on the top of the head corresponding to the biomechanical model of the IMU system (right of Figure 6). In Figure 6, bony landmarks (e.g., heel and knee) were used for the projected line-based yellow overlay of the skeleton so that the schematic avatar shows not the exact appearance but the posture of the body inside the image.

## 3. Application

Only the consideration of biomechanical principles allows the detailed description of effects such as integration into bottlenecks, passing of multidirectional streams or overtaking, especially at high densities. With the HTS the full-body motion in crowds is measurable. Open questions and hypotheses can now be studied: What are the variables influencing space requirements? Are arms lifted protectively in front of the body? How are the body parts rotated? How do adaptive step types depend on density? Why do short bottlenecks have a higher capacity than longer ones [65]? Does this have anything to do with a special step sequence when crossing a narrow passage? What is the reason for the controversially discussed “faster-is-slower” or “slower-is-faster” effect [66,67,68]? What happens inside a high motivated crowd, when clogging, side movements, stumbling or even falling occurs [69]?

Models which should be able to reproduce the above mentioned effects need more detailed descriptions of the dynamics inside crowds. These models have to consider, e.g., the step length or gait of bipedal movement and the orientation of the body in regard to the main movement direction to enhance the quality of simulation results [33,34,35,36]. For this enhancement the HTS enables the performance of appropriate experiments and the collection of required data.

In the following section, the data of a bottleneck experiment show examples of the possibilities one has with the newly introduced HTS.

### 3.1. Experiment

**Figure 7:**
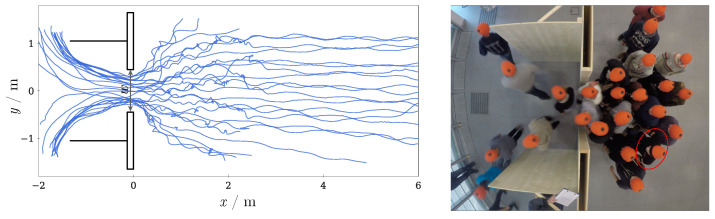
(**Left**) Schematic overview of the experimental setup for a bottleneck width of *w* = 1 m. The walls are drawn in black and the camera trajectories of all participants in blue. (**Right**) Overhead camera view of the corresponding experiment with people wearing orange caps with black dots. The red encircled person is wearing the IMU system.

The first experiments using the hybrid system were performed in a meeting room called rotunda with a size of 166 m^2^ at Forschungszentrum Jülich, Germany in 2018. The blinds were closed to eliminate changing lighting conditions and to prevent blending of people. The artificial lights at the ceiling were set to maximum intensity. The number of participants was 25 and consisted mainly of students and some staff members. A bottleneck of 0.13 m length and a variable width *w* constructed with wooden walls had been installed (see right of Figure 7). Behind the bottleneck, walls were placed on the left and right sides to prevent early turning of the participants. The space behind the bottleneck was sufficient to prevent a backlog of the flow through the bottleneck.

The bottleneck width varied between 0.7 m and 1 m with an increment of 0.1 m. For each width, two motivations were investigated: low (h-) and high (h0) motivation. The instructions to achieve the intended motivation were as follows:

h-: Participants were told that they had tickets (e.g., for a concert) with seat reservation and they would be able to reach their seat in time.
h0: Participants were told they had tickets without a seat reservation and thus would get a better seat if they entered earlier.

For statistical reasons, two runs were performed for each motivation and width.

In all experiments the same participant was wearing the MoCap system MVN Awinda from Xsens [37] (red circled person in the right picture of Figure 7). This person was a male, had a body height of 1.96 m and a shoulder width of 0.42 m and started each run from the same position in the line up area.

Two cameras had been installed overhead to obtain the trajectories of the participants as well as velocities and densities. The participants were wearing orange caps each with a black dot in the center to simplify the extraction of trajectories [7].

The participants were told to walk from a line up area towards the bottleneck and then gather directly in front of the bottleneck before walking through it after a signal was given.

A schematic overview of the setup is shown on the left of Figure 7. The figure shows a run with a bottleneck width of *w* = 1 m. The walls are drawn in black and the camera trajectories of all participants are visualized in blue. On the right of Figure 7, one camera view from overhead of the corresponding experiment is shown, which was used to extract the camera trajectories with help of the caps.

### 3.2. Results

The data obtained from the experiment were analyzed regarding the overall movement sequence and the influences of bottleneck width and motivation on the motion pattern, focusing on the combined analysis of the head position, velocity, foot position and joint angle of the pelvis. The analysis was intended to demonstrate the possibilities of the HTS and was not meant to be a comprehensive analysis, also because the data available were too limited to make a reliable general statement.

**Figure 8:**
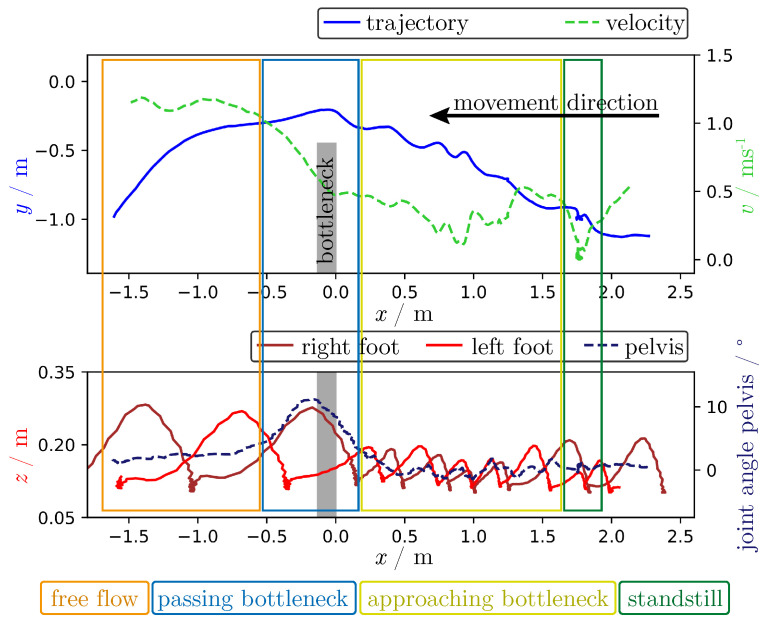
Trajectory of the head position in xy-plane (blue curve), velocity (dashed green), foot position in xz-plane (dark red for right and light red for left foot) and joint angle of pelvis (dashed dark blue) for a bottleneck experiment of *w* = 0.9 m width and high motivation, h0. The person wearing the MoCap system moved from right to left. Phases within the experiment marked with rectangles: standing still (green), stagnant movement to the bottleneck entrance with small steps (yellow), crossing the bottleneck (shown as a gray rectangle) with a rotated pelvis (cyan) and free flow with larger steps and free flow velocity after passing through the bottleneck (orange).

Figure 8 shows above mentioned parameters of the participant wearing the MoCap system walking through a bottleneck of 0.9 m width with high motivation. The upper diagram shows in blue the head trajectory f(t) in the xy-plane, while the person goes from right to left in accordance with a decreasing *x*-value.

A gray rectangle starting at x=0 indicates the position of the bottleneck. The upper diagram visualizes only the lower part of the experimental area so that only one side of the bottleneck wall can be seen.

The green dashed velocity v(t) is calculated as follows:

(7) $$v\left(t\right)=\frac{∥f(t+\Delta t)-f(t-\Delta t)∥}{2\Delta t}\;\mathrm{with}\;\Delta t=0.2\;s.$$

The lower diagram of Figure 8 shows the height above the ground (*z*-position) of both feet in dark and light red coming from the MoCap system. The sensor was mounted on top of the instep so that the height was larger than zero. Although the values are plotted along the *x*-axis and not along the main movement direction, the steps are clearly visible. The dashed dark blue line shows the joint angle of the pelvis, meaning the rotation of the hip with respect to the trunk. The *x*-axis and not the main movement direction is used for a direct comparison of the positional relation between both diagrams. The pelvis rotation and stride sequence were chosen because influences were expected from the bottleneck width and level of motivation.

The following describes the phases of the overall movement seen in Figure 8 while walking through the bottleneck (marked with colored rectangles):

dark green: Approximately 1.8 m in front of the bottleneck, the participant was standing still. The velocity was 0 ms^−1^ and the person’s feet were close to each other.
yellow: Between 1.8 m and 0.2 m in front of the bottleneck, the participant was walking towards the bottleneck, making small steps with variable length depending on the space available with fluctuating velocity. A periodicity of the steps for both feet and also tripping steps with both feet near to each other and tiptoeing directly in front of the bottleneck are visible. While the participant was making smaller steps, the transversal movement of the head from swaying increased, as can be observed in the trajectory, and also the joint angle of the pelvis was fluctuating from slow bipedal movement.
cyan: In front of the bottleneck, the participant was making small steps and then walked through the bottleneck with one large step. The participant was rotating the pelvis to be able to go through the bottleneck while other participants were walking through the bottleneck as well, decreasing the available space perpendicular to the movement direction.
orange: Having passed the bottleneck, step length and velocity increased. The joint angle of the pelvis stayed a bit above zero while circuiting the walls of the bottleneck (compare Figure 7).

**Figure 9:**
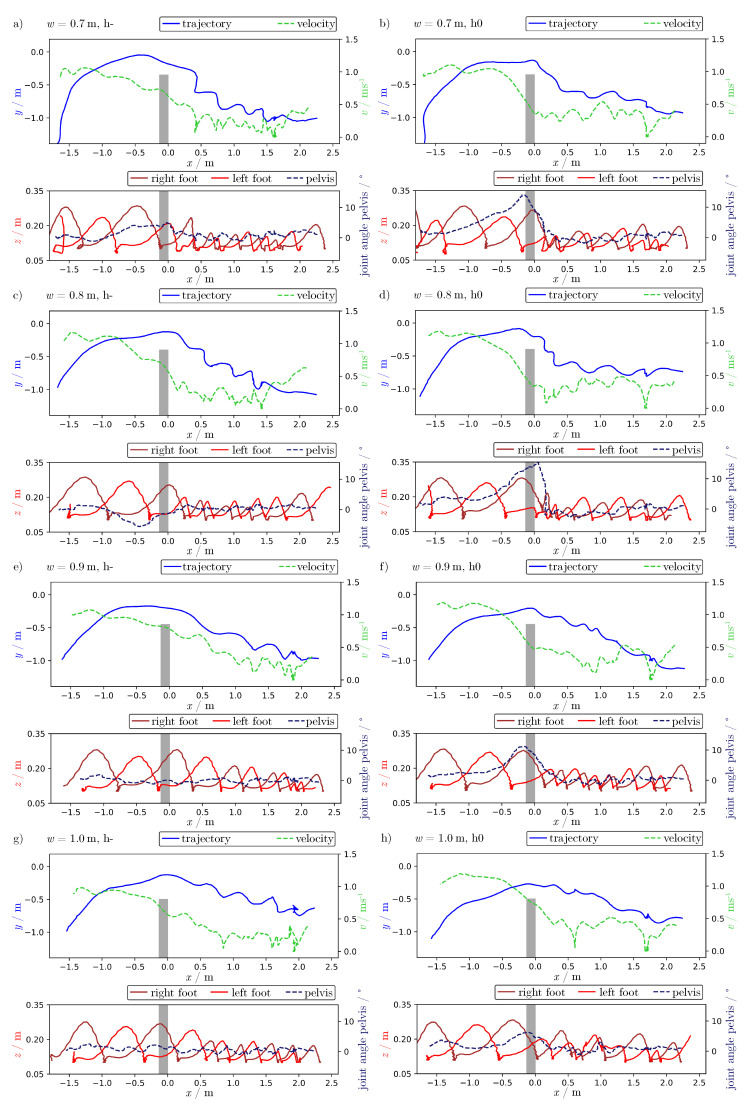
Head position (blue), velocity (dashed green), foot position (red) and joint angle of pelvis (dashed dark blue) for increasing bottleneck widths *w* (top to bottom) and low (h-, left) and high (h0, right) motivations: (**a**) 0.7 m, h-, (**b**) 0.7 m, h0, (**c**) 0.8 m, h-, (**d**) 0.8 m, h0, (**e**) 0.9 m, h-, (**f**) 0.9 m, h0, (**g**) 1.0 m, h- and (**h**) 1.0 m, h0.

Figure 9 shows the same diagrams for all varied parameters: for bottleneck widths of 0.7 m to 1.0 m (from top to bottom) and motivation levels h- (left) and h0 (right). This allowed us to test but not evaluate conclusively hypotheses of how bottleneck width and motivation influenced the studied characteristics of motion.

The following things are visible. The velocity in front of the bottleneck was temporary higher for high motivation due to a competitive contraction phase. The rotation angles of the pelvis while walking through the bottleneck was larger for high motivation. This joint angle was also larger for small bottleneck widths. Table 2 lists the maximum rotation angle of the pelvis while passing through the bottleneck, illustrating this dependency. Small steps correlate with low velocity. Smaller steps appear closer to the bottleneck for high motivation and small bottleneck widths. This can be interpreted as a sign of congestion directly in front of the bottleneck. The person wearing the MoCap system started at the same place left from the center of the bottleneck, and thus circuit the walls of the bottleneck also to the left. After passing through the bottleneck with a rotation of the pelvis to left regarding the direction of movement (positive angle), it can be observed that the rotation of the pelvis was slightly retained. In Figure 9c, the rotation while passing through the bottleneck is in contrast to the following side of movement (negative angle) and goes back to zero. For larger bottleneck widths and low motivation (Figure 9e,g), no congestion seemed to occur, because larger steps and no rotation while passing the bottleneck were visible. The temporal fluctuation is not discoverable, because all values are plotted along the *x*-axis.

## 4. Conclusions and Outlook

The use of IMU-based full-body motion capturing systems for experiments studying pedestrian dynamics inside crowds makes it possible to gain trajectories and information about body movements which cannot be extracted by a system needing a free line of sight to the tracked object, such as a camera system.

For the proposed hybrid tracking system, a camera-based system that tracks the head of a person and a MoCap system based on IMUs complete each other perfectly. The well-calibrated camera system is able to extract the absolute head position with high accuracy but without additional knowledge of the body movement, while the MoCap system is able to determine the relative full-body motion in detail but is prone to positional drift. The main idea for data fusion is using averaged synchronized positions of both systems to calculate the translational and rotational differences between the datasets and then using the transformed detailed MoCap data in combination with the smoothed head trajectory of the camera system. It has been shown that for data fusion, very accurate time synchronization is needed.

The fused data of the HTS opens up completely new possibilities for analyzing pedestrian dynamics inside crowds and, e.g., enables for the first time capturing and investigation of space requirements while passing a bottleneck, especially at high densities. The system allows linking any body motion to characteristics describing the situation of a person inside a crowd, such as the density or movements of other participants nearby, and also gives the possibility of bringing the motion in connection to other boundary conditions, such as the walls of pedestrian facilities.

A small bottleneck experiment showed some of the possibilities one gets with the newly introduced HTS. A combined analysis of the head position, velocity, foot position and joint angle of the pelvis pointed out the influences of bottleneck width and motivation on motion patterns. The hypotheses of decreasing step length and increasing rotation of the pelvis with decreasing bottleneck width and increasing motivation could be strengthened.

In order to be able to make reliable statements, more data are needed. At least the influences of age, gender and height should be taken into account. For this we bought 20 additional MoCap systems. With the correct positional integration of the MoCap data it is then possible to study the full-body interactions between people inside crowds. The positions of their bodies to and the interactions between each other could be investigated as well as the demand of space for protecting arms, shoulders, hip or feet. The above-mentioned small experiments were limited to the consideration of only a single person. For that concrete application, it would even have been enough to synchronize both systems in time and evaluate the data of each system separately from each other.

The quality of the used MoCap system from Xsens for itself has already been shown [61]. However, the contact between people that occurs in crowds, especially if highly motivated people are present, can cause single sensors to be moved externally. The influence of external impact on the quality of the results of the MoCap system and thus, how robust the biomechanical model is, has still to be determined.

Figure 6 gives a qualitative impression of the correctness. The possibility of the visualization of an overlay of a schematic avatar to the corresponding video has been integrated into the open source software PeTrack [70].

Using stereo cameras instead of monocular cameras would enable the detection of the 3D position of the head, including bobbing. This would increase the accuracy of the absolute position as input for the HTS. The influence on the HTS result has to be studied, because the local 3D movement is only taken from the IMU system. The influence of the stereo data on the smoothed main movement direction may be negligible.

During fusion, the head trajectory of the IMU data could be stretched or compressed to the overall length of the camera trajectory. The underlying biomechanical model stays and the data of the full body are shifted in total according to Equation (5). This could lead to errors, e.g., because the stepping uses zero velocity updates when a foot stays still. Such errors become visible through sliding people, where the floor-placed foot can slide during the stand still phase of the foot. This could be reduced by scaling the data of the whole body, e.g., using the relation between the length of the orientation vectors $∥r_{p}\left(t\right)∥/∥r_{u}\left(t\right)∥$.

Although the possibilities of the HTS raise the analysis of pedestrian dynamics to a new level, additional sensor data could widen the understanding even further. For example, eye trackers could give insights into the spatial perceptions and the operational principles of way finding, but also how far a person inside a crowd is looking ahead influencing his behaviour or to his feet for taking care to others. Psychophysiological parameters such as heart rate or skin conductance could be measured as well to link, e.g., the stress level to biomechanical parameters. Last but not least, sensors could measure the pressure between people inside crowds or on surrounding facilities, because injuries in dense crowds can be caused by high pressure.

## Figures and Tables

**Table 1:** Average distance $\bar{d_{\Delta t}}$ between the fused trajectory and the camera trajectory in the xy-plane for a given time offset Δt from the synchronous time. For the bold offset, the corresponding trajectories are shown in Figure 4.

Time Offset Δt / s	Average Distance $\bar{d_{\Delta t}}$ / cm
−0.02	1.02
**0**	0.86
0.02	1.02
**0.1**	2.87
0.2	4.28
**0.5**	6.61
**0.9**	3.13

**Table 2:** Maximum rotation angle of the pelvis while passing through the bottleneck ( 0.2 m before and after the wall forming the bottleneck).

Motivation	Bottleneck Width *w* / m			
	0.7 m	0.8 m	0.9 m	1.0 m
low (h-)	4.7°	4.5°	1.3°	2.1°
high (h0)	13.9°	15.3°	11.1°	6.2°

## Data Availability

This methodical paper focuses on a newly introduced approach for fusing sensor data. Supplementary data of mentioned experiments will be published after finishing the content-related data analysis at the data archive [31].

## Abbreviations

   The following abbreviations are used in this manuscript:

IMU	Inertial Measurement Unit
GPS	Global Positioning System
UWB	Ultra Wideband
TOA	Time Of Arrival
MoCap	Motion Capturing
HTS	Hybrid Tracking System
